# Understanding the Impact of Male Circumcision Interventions on the Spread of HIV in Southern Africa

**DOI:** 10.1371/journal.pone.0002212

**Published:** 2008-05-21

**Authors:** Timothy B. Hallett, Kanwarjit Singh, Jennifer A. Smith, Richard G. White, Laith J. Abu-Raddad, Geoff P. Garnett

**Affiliations:** 1 Department of Infectious Disease Epidemiology, Imperial College London, London, United Kingdom; 2 Bill & Melinda Gates Foundation, Seattle, Washington, United States of America; 3 Infectious Disease Epidemiology Unit, London School of Hygiene and Tropical Medicine, London, United Kingdom; 4 Vaccine and Infectious Disease Institute, Fred Hutchinson Cancer Research Center, Seattle, Washington, United States of America; Norwegian Knowledge Centre for the Health Services, Norway

## Abstract

**Background:**

Three randomised controlled trials have clearly shown that circumcision of adult men reduces the chance that they acquire HIV infection. However, the potential impact of circumcision programmes – either alone or in combination with other established approaches – is not known and no further field trials are planned. We have used a mathematical model, parameterised using existing trial findings, to understand and predict the impact of circumcision programmes at the population level.

**Findings:**

Our results indicate that circumcision will lead to reductions in incidence for women and uncircumcised men, as well as those circumcised, but that even the most effective intervention is unlikely to completely stem the spread of the virus. Without additional interventions, HIV incidence could eventually be reduced by 25–35%, depending on the level of coverage achieved and whether onward transmission from circumcised men is also reduced. However, circumcision interventions can act synergistically with other types of prevention programmes, and if efforts to change behaviour are increased in parallel with the scale-up of circumcision services, then dramatic reductions in HIV incidence could be achieved. In the long-term, this could lead to reduced AIDS deaths and less need for anti-retroviral therapy. Any increases in risk behaviours following circumcision , i.e. ‘risk compensation’, could offset some of the potential benefit of the intervention, especially for women, but only very large increases would lead to more infections overall.

**Conclusions:**

Circumcision will not be the silver bullet to prevent HIV transmission, but interventions could help to substantially protect men and women from infection, especially in combination with other approaches.

## Introduction

In response to improved surveillance data, the Joint United Nations Agency on AIDS (UNAIDS) has recently revised official estimates of the numbers living with HIV worldwide [1]. These figures show that HIV prevalence in Africa probably peaked in the late 1990s. However, in most countries this is due to an increase in AIDS deaths coinciding with the epidemic predominantly moving to lower risk individuals [2], [3], [4], [5], [6]. It has been suggested that a significant corner has been turned in the fight against the HIV epidemic [7], but an incidence rate where 2.8 million men, women and children newly infected each year in sub-Saharan Africa is unacceptable [1]. Our failure to reduce incidence more substantially requires a re-examination of what interventions, singly or in combination, are best suited to reverse the HIV pandemic.

Significant scientific advances have been made in understanding how heterosexual transmission can be limited. First, the potential impact of behaviour change was indicated in Uganda [4], and later Zimbabwe [8]. Trials in the 1990s showed that, whilst useful early on, improved bacterial STI management is unlikely to substantially reduce HIV incidence in mature HIV epidemics [9], [10], [11], [12], [13], [14]. In the last year, a population level behaviour change intervention was found to be ineffective [15] and risk compensation and low adherence may have contributed to no effect being found in trials to prevent HIV infection through diaphragm use [16] and herpes treatment [17], respectively. This tally adds to over 30 randomized clinical trials that failed to show efficacy or effectiveness in reducing HIV incidence [18]. However, three recent randomised controlled trials of adult male circumcision uniformly found that circumcised men are 60% less likely to be infected than others [19], [20], [21]. The World Health Organization (WHO) and UNAIDS have recommend that circumcision be considered as part of intervention programmes in high-prevalence settings [22], [23].

Although trials can provide definitive evidence on the impact of interventions at the individual-level [24], they do not provide information on how incidence might be reduced across populations, which is the primary concern in public health [25], [26]. No further trials are planned, so in this article, we have used existing data from trials and field studies in a mathematical model to help address some of the most pressing questions surrounding male circumcision. This should help ground planning and decision-making in an evidence-based framework [27], [28]. The programmatic issues which we have addressed are:

1. What is the likely net impact of circumcision interventions?
2. How will circumcision interventions interact with existing interventions?
3. Is there the potential for perverse impacts on HIV spread from scale up of circumcision?

## Methods

**Figure 1:**
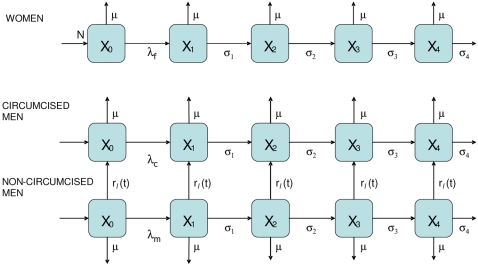
Flow diagram of model. The model population is divided into females, circumcised males and non-circumcised males. In each group, *X_0_* is the number of individuals not infected, *X_1_* is the number with acute HIV infection, *X_2_* is the number with latent infection, *X_3_* is the number of people in a stage shortly prior to AIDS and *X_4_* is the number with AIDS. The rate of progression between these stages of infection is given by σ_1_, σ_2_ and σ_3_ and the rate of death for those with AIDS is σ_4_; on average, individuals have acute infection for four months, latent infection for eight years, ‘pre-AIDS’ for twelve months and AIDS for six months. The rate of incidence among females, circumcised and non-circumcised men are represented by *λ_f_*, *λ_c_* and *λ_m_* respectively. The intervention is simulated by moving non-circumcised men into the group of circumcised men, at a rate which can be different for men who are infected from those who are not or different in the different sexual activity groups (not shown): *r_s_*(*t*).

A simple deterministic model of the heterosexual spread of HIV was developed (Figure 1) following established methods [29], [30], [31]. This type of model has been applied to many epidemiological problems previously, including predicting the impact of anti-retroviral therapy [32], [33], [34], interpreting trends in HIV prevalence and quantifying resource needs to tackle the epidemic [3], [35], [36]. Technical details are available in the online appendix (Text S1).

Published data from eastern Zimbabwe [8], [37] were used to inform the parameters specifying sexual behaviour, but the broad behavioural patterns are similar to reports in other settings [38], [39], [40]. In representing the observed heterogeneity in the number of sexual partners, men and women were stratified into risk groups that form different numbers of sexual partnerships. Those in the higher risk groups tend to form more partnerships, but each of these partnerships comprises fewer sex acts and condom use is greater. Men and women form partnerships so that it is more likely that high risk individuals form partnerships with one another. Based on observational data from a longitudinal study in rural Uganda [41], the course of infection is represented by individuals progressing through several stages: acute infection (short duration, high infectiousness), latent infection (long duration, low infectiousness) and pre-AIDS (short duration, high infectiousness). After pre-AIDS, a fraction of individuals develop full-blown AIDS and die, whist others start anti-retroviral therapy (ART) and survive for eight years with very low infectiousness. In our model, the fraction of individuals that can start treatment increases from 0% two years before the circumcision intervention starts (which we take to be approximately equal to calendar year 2005) to 28% within two years (i.e. 2007) – this is typical for sub-Saharan Africa [42]. We assume that ART coverage will plateau at 90% by 2020. If some risk groups suffer greater AIDS-related mortality than others, the model allows individuals to move between groups so that the overall mean and distribution of risk in the population is held constant.

Uncircumcised men can be circumcised in the intervention and it is assumed that circumcised men are 60% less likely to acquire infection each time they are exposed [19], [20], [21], [43]. The rate at which men are circumcised in the model is such that the eventual level of coverage of circumcision (fraction of men circumcised) is reached within 5 years of the intervention starting. In most simulations no effect on male-to-female transmission is assumed, despite some conflicting observational evidence that it may be reduced [44], [45], [46], [47], [48]. In some simulations it is assumed that there is short period of wound healing (2 months) immediately following the operation, during which time most men are not sexually active but the chance of transmission to women per sex act may be elevated [49].

## Results

### What is the likely net impact of circumcision interventions?

In the model it is assumed that the individual circumcised man has approximately 60% less risk of infection in each unprotected sex act with infected women. However, the chance that a man becomes infected will depend on his pattern of exposure to HIV. For instance, uninfected men in sero-discordant partnerships, or men with many sexual partners that do not use condoms, are likely to become infected whether or not they are circumcised because their high level of exposure will overwhelm the partial, if substantial, protection afforded. On the other hand, circumcised men in shorter relationships, or that regularly use condoms, will gain a proportionately greater benefit if exposed.

**Figure 2:**
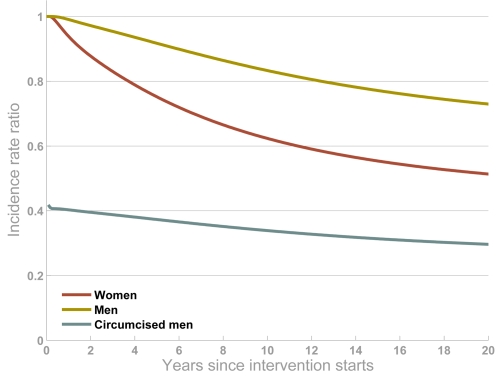
Impact of the intervention among women (red line), uncircumcised men (yellow line) and circumcised men (blue line) (90% circumcision coverage achieved). The output is the ratio of HIV incidence when the intervention is simulated relative to the projection with no intervention. In these simulations, the operation wound is assumed to heal instantaneously.

Although the intervention only directly protects circumcised men, there is an indirect benefit for women and uncircumcised men (Figure 2). Since they are at less risk of infection, HIV prevalence will decline among circumcised men and so, over time, their partners (and their partners' partners, and so on) will also be at less risk. Then, as prevalence gradually declines among women, the circumcised men also start to receive an indirect benefit of the intervention. Eventually, therefore, the effectiveness of the intervention for circumcised men actually exceeds the measured 60% biological efficacy (Figure 2).

**Figure 3:**
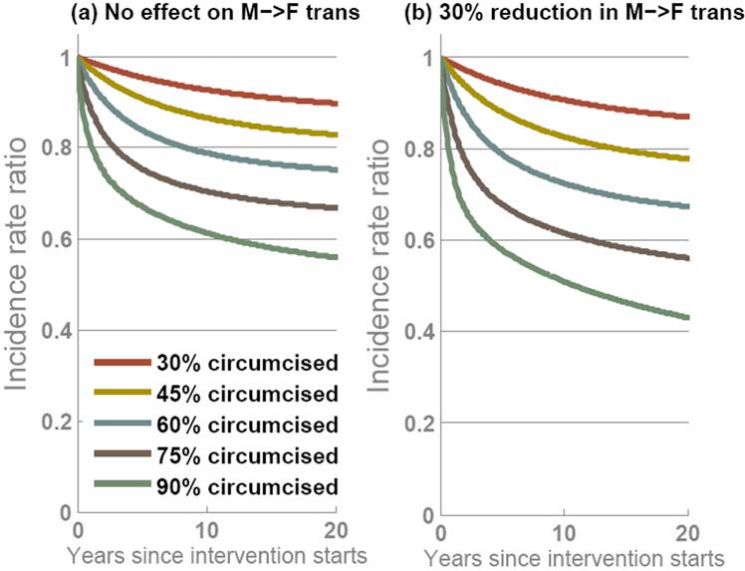
Projected impact of male circumcision interventions over time with different levels of coverage achieved if, (a) circumcised men are 60% less likely to get infected but there is no effect on male-to-female transmission; and, (b) circumcised men are 60% less likely to get infected and circumcised men are 30% less likely to transmit infection. In each panel, five epidemic projections show circumcision interventions with 30% (red line), 45% (yellow line), 60% (blue line), 75% (brown line) or 90% (green line) of men being circumcised. The output is the ratio of HIV incidence when the intervention is simulated relative to the projection with no intervention. Endemic HIV prevalence before the intervention is 23%.

The overall impact of circumcision interventions achieving different levels of coverage is examined in Figure 3. The relationship between coverage and overall impact at the population level is strong and non-linear, meaning that marginal increases in coverage lead to greater marginal gains in preventing new infections. With no effect on male-to-female transmission assumed, and 50% of men circumcised, once a new endemic prevalence is reached after 15–20 years, incidence across men and women is reduced by ∼20% (Figure 3(a)). However, it will take time for that full effect to be realised. Although the direct effect of reduced incidence in the circumcised men will almost immediately follow healing, reductions in incidence among women and uncircumcised men rely on prevalence declining among circumcised men. Since median survival with HIV infection is approximately 10 years, these indirect effect of the interventions emerge gradually over decades. This means that cumulative measures of the impact of the epidemic, which include infections prior to when the full effect is exerted, provide a less substantial indication of the effectiveness of this intervention [50]. Furthermore, over the long-term, interpreting the reduction in the number of infections due to the intervention as ‘infections averted’ is not straight forward as faster population growth (by reducing the effects of AIDS-related mortality and sub-fertility) can contribute to greater numbers of infections despite the rate of incidence being reduced.

The benefit to women is augmented if circumcised men are also less likely to transmit the infection [46] (Figure 3(b)). In this case, the indirect effect also emerges more quickly as women are put as less risk of infection immediately after men are circumcised. The strength of this additional protective effect for women depends on the fraction of men that are circumcised, and so the relationship between uptake of the intervention and the population-level impact becomes steeper, and the marginal gains in extending coverage are even greater.

### How will circumcision interventions interact with existing interventions?

**Figure 4:**
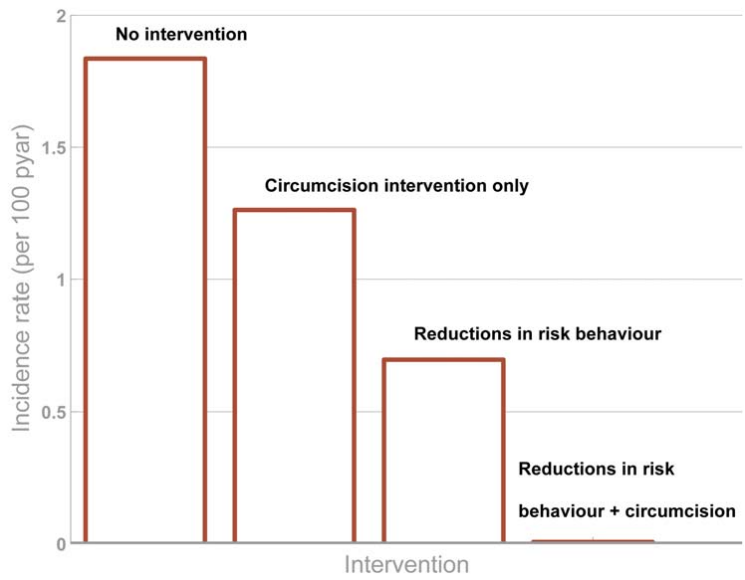
Interaction of circumcision interventions with existing behaviour change programmes. Four epidemic projections show: (i) no interventions, (ii) circumcision intervention with 90% coverage, (iii) a behaviour change intervention that leads to an average 30% reduction in partner change rate and 30% increase in condom use with casual partners, (iv) both the circumcision intervention and the behaviour change intervention. The output is HIV incidence per 100 person-years at risk (pyar). The time-scale relates to years since the *circumcision* intervention starts. (Note: Unlike in other simulations, here no compensation is made for the potential effects of AIDS mortality modifying the risk distribution in the population (see text S1 for details)).

Other models have indicated that with high coverage and with circumcised men less likely to transmit infection to women then circumcision could be used to eventually eliminate HIV [51]. However, in our analysis this does not appear to be possible, although the intervention does make HIV infectious spread unsustainable in some lower risk groups. A key result from early studies of infectious disease epidemiology [52] is that several interventions tend to operate synergistically (the specific contribution of alternative types of intervention will be determined by the epidemiological context [53]). That is, a circumcision intervention applied at the same time as other behavioural changes take place will lead to a much greater impact than would be expected on the basis of either in isolation. Figure 4 shows the incidence rate following different kinds of intervention: a circumcision intervention, a behaviour change intervention and both combined. In combination, the two interventions can drive the HIV epidemic to much lower levels.

**Figure 5:**
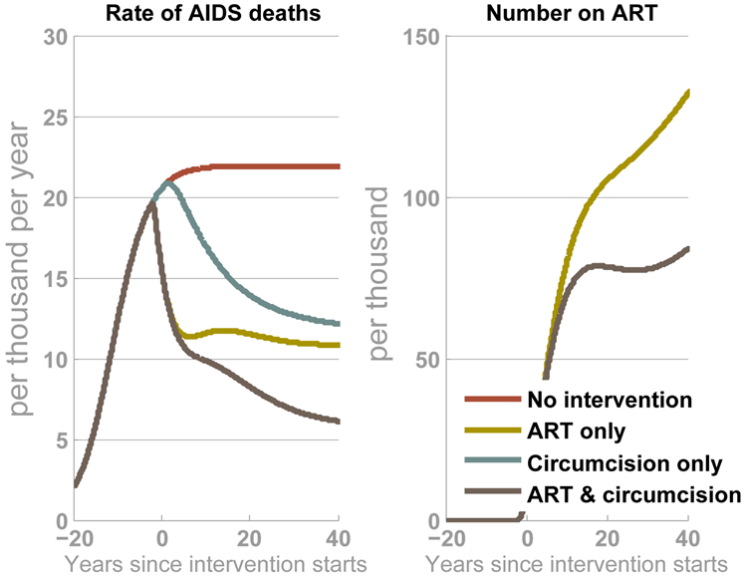
Comparison of circumcision interventions and ART. Four epidemic projections show (i) no interventions (red line), (ii) ART provided to up to 90% of those in need (yellow line), (iii) circumcision intervention with 90% coverage (blue line), (iv) both the ART and the circumcision intervention (brown line). The outputs are the annual rate of AIDS death (per 1000) and the number receiving ART (per 1000 population when the intervention starts). The time-scale relates to years since the *circumcision* intervention starts. The fraction of individuals that can start treatment increases from 0% two years before the circumcision intervention starts (which we take to be approximately equal to calendar year 2005) to 28% within two years (i.e. 2007) – this is typical for sub-Saharan Africa [42]. We assume that ART coverage will plateau at 90% by 2020.

Throughout southern Africa, ART programmes are being rapidly scaled-up [1], [42] and ART will interact with circumcision interventions. ART, by itself, is not likely to lead to substantial changes in incidence unless treatment can be initiated before individuals reach advanced disease [34], [54], [55]. However, the number of adult AIDS deaths is substantially reduced immediately as ART programmes are scaled-up (Figure 5). The circumcision intervention does not lead to fewer AIDS deaths in the short-term, but in the longer-term a reduction of comparable magnitude is achieved (Figure 5). In combination, ART and circumcision programmes lead to great reductions in deaths and infections, in both the short and long-term. Furthermore, interventions like circumcision that reduce the number of infections today, will also curb the growing demand for ART and treatment for other opportunistic infections tomorrow, leading to considerable long-term cost savings [36], [56].

### Is there the potential for perverse impacts on HIV spread from scale up of circumcision?

**Figure 6:**
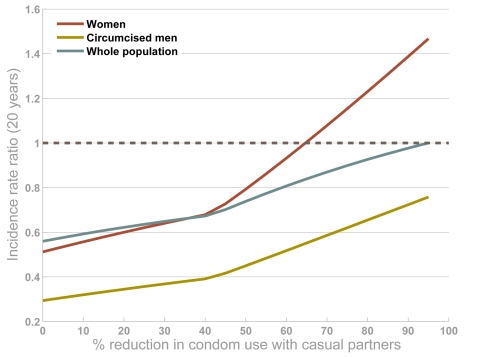
The impact of risk compensation by circumcised men. Men that are circumcised in the intervention are assumed to reduce the chance they use condom with casual partners (originally 0.6 [8], [37]) by between 0 (no change) and 100% (no condom use at all with causal partners). The output is the ratio of HIV incidence twenty years after the intervention starts among women (red line), circumcised men (yellow line), and the population overall (blue line) when the intervention is simulated relative to the projection with no intervention. A value greater than 1 indicates that incidence is higher with the intervention. It is assumed that 90% of men are circumcised in the intervention.

Two major concerns have surrounded plans to offer circumcision as an intervention – behavioural risk compensation and transmission from infected men resuming sex before the wound heals. Risk compensation is the phenomenon whereby those who have taken protective steps offset the benefit with other behavioural changes. One typical example is people using sunscreen spending more time in the sun [57]. In the context of male circumcision, risk compensation could include many types of behavioural change, including increased numbers of casual partners and less condom use. The effect of risk compensation is complicated because circumcised men with increased risk are not only putting themselves at more risk of infection but also their sexual partners. In fact, the high degree of protection afforded by circumcision means that only extreme levels of risk compensation would lead to circumcised men being at more risk of infection than otherwise (Figure 6). But, more moderate levels of risk compensation could lead to more women becoming infected. In our simulations, if men are ∼60% less likely to use condoms with casual partners after being circumcised there could be more infections among women (Figure 6). Over the whole population, however, incidence would still be reduced, by ∼16%. If men reduced condom use by 90%, then incidence among women could increase by 40% and lead to more infections overall (Figure 6). The chance that increased risk behaviour leads to more infections is greater in low-level epidemics, where changes in incidence are more sensitive to increases or decreases in risk behaviour. In these simulations, we assume that 90% of men are circumcised in the intervention but with lower uptake, the chance of perverse outcomes is smaller. The inflexion shown in Figure 6 represents lower-risk groups becoming able to support chains of transmission when condom use is less frequent [31], [58].

**Figure 7:**
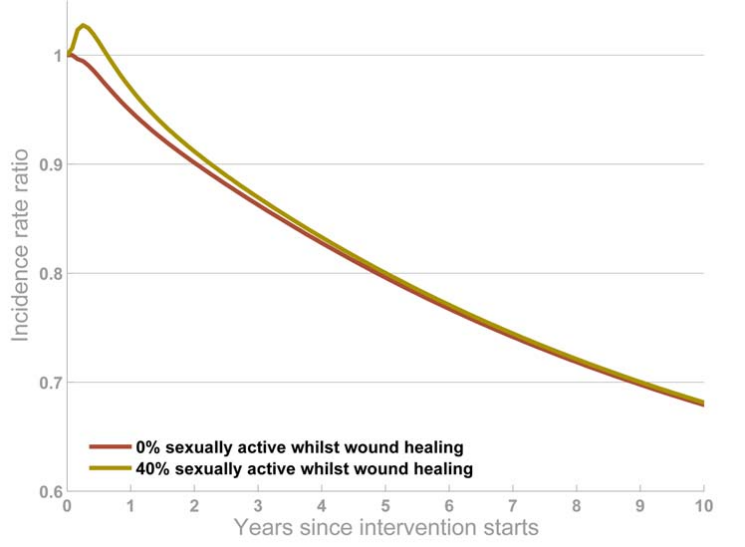
The impact of the wound healing period and circumcising infected men. The projections show the impact of a circumcision intervention with 90% of men being circumcised if it is assumed that the chance of transmission from circumcised men to women is twice as high during the two-month healing period, and 0% of circumcised men (red line) or 40% of men (yellow line) are sexually active whilst the wound heals (yellow line). The output is the ratio of HIV incidence when the intervention is simulated relative to the projection with no intervention.

Another concern is that HIV transmission from infected circumcised men might be higher in the period when the wound is healing, if they resume sexual activity. There was a indication (not statistically significant) that this was the case in a trial in Uganda investigating male-to-female transmission that was stopped early [49]. To explore the potential impact of this at the population level, we simulated the impact of the interventions assuming that HIV-infected men who are circumcised are twice as likely to transmit the infection as uncircumcised men during a two-month healing period. This is approximately equal to the relative transmission effect observed in the trial but it is unlikely that wounds would normally take that long to heal. We also pessimistically assume that 40% of men resume sexually active immediately after the operation – in the trial, less than 20% of men resumed sexual activity sometime before the wound had fully healed [49]. These pessimistic assumptions had very little influence on the outcome of the intervention among women and more widely (Figure 7). If the circumcision wound does increase transmission *and* men are sexually active before the wound heals, incidence may rise slightly during the first years of the intervention (in our model, many men are circumcised at the same time at the start of the intervention). However, the eventual impact of the intervention is much the same as if it is assumed that transmission is not increased or men do not resume sexual activity prematurely.

## Discussion

Our primary finding is that circumcision alone will not be the ‘silver bullet’ that halts the HIV epidemic. A more likely scenario is for incidence to be eventually reduced by 25–35% if high coverage levels are achieved (Figure 3). It has previously been suggested that the reduction in rate of HIV transmission from female-to-circumcised-male of 60% is comparable to a vaccine delivered to both men and women of about 37% efficacy, if all men are circumcised [59]. Our model is good agreement with that finding, but although acceptability of male circumcision has been reported at promisingly high levels (*circa* 50% in many settings [60]), complete coverage seems implausible. With lower coverage, a weaker net effect is projected (Figure 3).

The indirect benefit of circumcision interventions to women (and uncircumcised men) is mediated by reduction in HIV prevalence among their circumcised male sexual partners (and partners' partners). It is slower to emerge because the long survival time with HIV means that prevalence declines gradually. Furthermore, since it comes via the sex partner network, its extent is extremely difficult to predict because sexual behaviour is multi-faceted [61], sometimes incompletely reported [62] and because the pattern of transmission is also strongly linked to higher-order sexual network properties [63], [64]. Some simple models may fail to capture the full extent of this indirect effect and under-estimate the total impact of the intervention [51], [65]. Similarly, analyses that measure the impact of circumcision over only short periods (<10 years) will not quantify the full benefit of the intervention [51]. In addition, because of the long term consequences of the intervention it is very important that the value of future benefits is appropriately discounted in economic analyses. For these reasons, other studies have shown that when a short-term time-frame is considered, quicker scale-up of services can substantially increase the overall impact [27], [66], [67].

The impact of any intervention depends on the existing patterns of risk and transmission in the population: the epidemiological context [53]. Epidemics are sustained if the chain of transmission (one individual infecting another) is maintained. Generally, in communities with low risk, that chain is fragile and may be broken by small biological or behavioural changes. If there is more risk, the same changes have less impact because the chain is still maintained. However, the smaller absolute number of infections means that, in such settings, more operations may be required to achieve the same number of infections averted. Thus, in general, the potential proportional impact of interventions on the epidemic is greater in low-prevalence, low-circumcision groups. The lowest cost per infection averted, in contrast, will be achieved in higher prevalence communities. However, since many alternative patterns of risk can lead to the same endemic prevalence level, it not possible to accurately judge the impact of the intervention using only that information. For instance, “low” prevalence in a country can signify either a core of high-risk behaviour with the rest at no risk of infection (where the intervention could have little impact), or a more even distribution of moderate risk throughout the population (where the intervention could substantially contribute to arresting transmission).

In combination with other behavioural changes, the impact of circumcision interventions could be much greater. At the individual-level, men that protect themselves with condoms will get a disproportionately greater protective benefit from circumcision, and at the population-level, synergies between interventions will amplify the reduction in incidence. To avoid wasting resources and a unique opportunity, circumcision programmes must be accompanied by a renewed and vigorous focus on behaviour change [4], [8], [68]. Circumcision programmes will also operate well alongside ART programmes. The modest reduction in new infections due to ART will be supplemented by reductions due to circumcision. That will lead to a reduced demand for ART in the future and, in the meantime, deaths due to AIDS will fall substantially.

Risk compensation could dent the impact of the intervention, so it will be especially important for safe-sex messages to be reinforced for men being circumcised. Increased risk behaviour could undermine derived benefits for women especially, but net increases in incidence (among women or the population overall) are only associated with very great increases in risk. Data from the three randomised trials [19], [20], [21] and another cohort study [69] did not find evidence for such large changes in risk following circumcision. Being able to avoid using condoms or having more sexual partners are not among the reported reasons for getting circumcised [70].

The conflicting evidence on the benefits of circumcising infected men [44], [45], [46], [47], [48], including the chance that transmission is increased if men resume sex before the wound has healed [49], and our modelling results leads to interesting ethical dilemma. There is clear advantage in circumcising infected men if the operation does reduce the chance of male-to-female transmission, but even if it does not and transmission is greatly increased during the healing period, the impact of the intervention for the population is not considerably reduced. However, individual specific women may be placed at greater risk. This has to be considered against the potential reduction in uptake if HIV-testing is a pre-requisite for being circumcised (necessary to avoid circumcising any infected men). On balance, in the interests of doing no harm, it is likely that the protection of the individual will outweigh the protection for the population. However, our modelling shows that this is borne out of a concern for the individual not the population.

We have explored the sensitive of our findings to the parameters specifying the pattern of heterosexual HIV transmission and the biological effect of circumcision and we expect that our conclusions will be applicable generally to the mature generalised epidemics of southern Africa. The precise impact of interventions will be determined by many local factors, including the epidemiological context, the level and pattern of uptake of the intervention, the biological effect of circumcision and the degree of risk compensation [53]. It will be important for quantitative projections to be tailored to the local situation and to incorporate as much data as possible on the historic epidemic trends and sexual behaviour. We also recognise that the impact of the interventions will be lower if the biological effect of circumcision is less than was found in the carefully conducted and well managed trials [19], [20], [21].

It is important in modelling work to establish the influence of model structure on the results – that is, whether the conclusions drawn are linked to the formulation of a particular model. Our findings are in close quantitative agreement with different types of model that have focussed on other settings and employed alternative analytic techniques [51], [59], [65], [71], [72]. Future work will aim to identify ways in which the impact of the intervention could be maximised, quantify uncertainty in projections and explore different techniques for predicting the impact of interventions, from micro-simulation to tractable analysis [71]. Mathematical modelling must build upon the gold-standard evidence from the randomised controlled trials to provide both qualitative understanding and detailed quantitative predictions to support the decision-making processes that are now underway.

## Supporting Information

Text S1 Technical Appendix
(0.14 MB PDF)
