# Supplementary material for: Understanding the Impact of Male Circumcision Interventions on the Spread of HIV in Southern Africa
Source: PLoS One. 2008 May 21;3(5):e2212. doi: 10.1371/journal.pone.0002212 (PMC2387228; doi:10.1371/journal.pone.0002212)
Supplement: Text S1 — Technical Appendix (0.14 MB PDF) [file pone.0002212.s001.pdf]

Text S1: Circumcision Model Technical Specification

The model is described in the following four sections: (i) Differential equations; (ii) Simulating the intervention; (iii) Calculating the force of infection; (iv) Parameterisation; and, (v) Model outputs.

(i) Differential equations

The model is defined by a set of ordinary differential equations which are solved numerically using Euler's method with a time-step of 1/12 years. The state variables are given by $X^{s}_{k,l}$: *s* is the infection-status (1= susceptible; 2= acute infection; 3= latent infection; 4= pre-AIDS; 5= AIDS; 6= on ART; 7= failed ART), *k* is gender/circumcision status (1= female; 2= uncircumcised male; 3= circumcised male wound healing; 4= circumcised male wound healed) and *l* is the sexual activity risk group (1= (highest risk), 2 and 3= (lowest risk)).

The ordinary differential equations describing changes in the state variable over time are:

$$\frac{dX^{1}_{1,l}}{dt} = \phi_{1,l}\frac{(\mu+\alpha)}{2}X^{\bullet}_{\bullet,\bullet} - \lambda_{1,l}X^{1}_{1,l} - \mu X^{1}_{1,l}$$

$$\frac{dX^{1}_{2,l}}{dt} = (1-h(t))\phi_{2,l}\frac{(\mu+\alpha)}{2}X^{\bullet}_{\bullet,\bullet} - \lambda_{2,l}X^{1}_{2,l} - \mu X^{1}_{2,l}$$

$$\frac{dX^{1}_{3,l}}{dt} = h(t)\phi_{3,l}\frac{(\mu+\alpha)}{2}X^{\bullet}_{\bullet,\bullet} - \lambda_{3,l}X^{1}_{3,l} - \mu X_{3,l}$$

$$\frac{dX^{2}_{k,l}}{dt} = \lambda_{k,l}X^{1}_{k,l} - (\mu+\sigma_2)X^{2}_{k,l}$$

$$\frac{dX^{3}_{k,l}}{dt} = \sigma_2 X^{2}_{k,l} - (\mu+\sigma_3)X^{3}_{k,l}$$

$$\frac{dX^{4}_{k,l}}{dt} = \sigma_3 X^{3}_{k,l} - (\mu+\sigma_4)X^{4}_{k,l}$$

$$\frac{dX^{5}_{k,l}}{dt} = (1-a(t))\sigma_4 X^{4}_{k,l} - (\mu+\sigma_5)X^{5}_{k,l}$$

$$\frac{dX^{6}_{k,l}}{dt} = a(t)\sigma_4 X^{4}_{k,l} - (\mu+\sigma_6)X^{6}_{k,l}$$

$$\frac{dX^{6}_{k,l}}{dt} = a(t)\sigma_6 X^{6}_{k,l} - (\mu+\sigma_7)X^{7}_{k,l}$$

In the absence of AIDS, the population grows exponentially at a rate α. The fraction of men and women starting sexual activity in each risk group is $\phi_{k,l}$ (such that $\phi_{k,\bullet}=1$). $\lambda_{k,l}$ is the force of infection for individuals of that gender and sexual activity group. $h(t)$ is the fraction of men starting sex at time t that have already been circumcised.

To simulate the intervention moving uncircumcised adult men into the circumcised classes, the following additional terms are included:

$$\text{replace } \frac{dX_{2,l}^{s}}{dt} \text{ with } \frac{dX_{2,l}^{s}}{dt} - r_{s,l}X_{2,l}^{s}$$
$$\text{replace } \frac{dX_{3,l}^{s}}{dt} \text{ with } \frac{dX_{3,l}^{s}}{dt} + r_{s,l}X_{2,l}^{s} - \frac{1}{w}X_{3,l}^{s}$$
$$\text{replace } \frac{dX_{4,l}^{s}}{dt} \text{ with } \frac{dX_{4,l}^{s}}{dt} + \frac{1}{w}X_{3,l}^{s}$$

Here, $r_{s,l}$ is the rate at which uncircumcised men of that sero-status in that risk group are circumcised in the intervention and w is the mean duration of wound healing.

The fraction of individuals progressing to AIDS that start treatment is given by a(t). This value changes over time according to:

$$a(t) = a_{\max}\left[1 - \exp\left(a_{rate}\left(t - a_{start}\right)\right)\right]$$

Where $a_{\max}$ is the maximum level of coverage achieved, a_{start} is the time in the simulation when ART coverage starts to increase and a_{rate} is the rate in increase in coverage.

The rate of deaths due to AIDS include those dying of AIDS and those dying after ART has failed: $\sigma_5 X_{\bullet,\bullet}^{5} + \sigma_7 X_{\bullet,\bullet}^{7}$.

Over the course of epidemics, some risk-groups may suffer greater AIDS-related mortality than others, leading to progressive changes in distribution of risk in the population. In the model, this can be allowed and it is assumed that individuals cannot change risk-group during their lifetime and the proportion of individuals entering each risk group is constant over time (this assumption is labelled *R*=0). Alternatively, the model can counteract that change and allow individuals to move between risk-groups in such a way that the fraction of adult men and women in each risk-group remains constant over time (this assumption is labelled *R*=1) – this is the default assumptions used in the simulations presented. This is simulated in the following way:

$$\text{for } l = 1,2:$$
$$G_{k,l} = \phi_{k,l} X^{\bullet}_{k,\bullet} - X^{\bullet}_{k,l}$$
$$T^{s}_{1,l} = G_{1,l} \frac{X^{s}_{1,l+1}}{X^{\bullet}_{1,l+1}}$$
$$T^{s}_{k,l} = G_{k,l} \frac{X^{s}_{k,l+1}}{\sum_{k=2}^{4} X^{\bullet}_{k,l+1}} \text{ for k} > 1$$
$$X^{s}_{k,l} \rightarrow X^{s}_{k,l} + T^{s}_{k,l}$$
$$X^{s}_{k,l+1} \rightarrow X^{s}_{k,l+1} + T^{s}_{k,l}$$

(ii) Simulating the intervention

The overall rate at which men are circumcised is $v(t)$,which increases from zero linearly over a period τ to its maximum value $v_{\max}$. The fraction of men starting sex that are already circumcised ($h(t)$) follows the same pattern (with maximum fraction $g_{\max}$). Holding the overall rate at which men are circumcised constant, the rate at which in particular groups are circumcised is varied in the following way.

$$r_{s,l} = \left(1 - \frac{p(t)}{p_{\max}}\right) F_{s,l} \frac{v(t)}{\sum_{l}\sum_{s}\left(F_{s,l} \frac{X^{s}_{2,l}}{X^{\bullet}_{2,\bullet}}\right)}$$

The first term in this equation limits the total fraction of the male population that are circumcised to less than p_{max}. The fraction circumcised at time t is: $p(t) = \frac{\sum_{k=3}^{3} X^{\bullet}_{k,\bullet}}{\sum_{k=2}^{3} X^{\bullet}_{k,\bullet}}$.

$$F_{s,l} = f_s . f_l$$
$$f_s = T_{HIV} \text{ if s} > 1$$
$$f_s = 1 \text{ otherwise}$$
$$f_l = T_{RISK} \text{ if } l < 3$$
$$f_l = 1 \text{ otherwise}$$

T_{HIV} and T_{RISK} are the relative chance that uncircumcised men that are infected with HIV or in the higher two risk groups (l=1 or 2) get circumcised in the intervention, relative to uninfected and low risk men, respectively.

(iii) Calculating the force of infection

The force of infection is calculated on the basis of the partner change rate of individuals, HIV prevalence among their sexual partners, the number of sex acts in each partnerships and the use of condoms.

Individuals in each gender and risk-group form partnerships at a set rate: $c_{k,l}$, which is parameterised using a mean rate of partner change for that gender ($\bar{c}_k$) and two parameters that give the relative partner change rate for those in the highest ($\varpi_{k,1}$) and next-highest ($\varpi_{k,2}$) risk groups relative to those in the lowest risk group (for convenience, we also define $\varpi_{k,3} = 1$). The partner change rates can be then be calculated:

$$c_{k,l} = \varpi_{k,l} \frac{\bar{c}_k}{\sum_l \phi_{k,l} \varpi_{k,l}}$$

Men and women form partnerships in such a way that a fraction ε of their partnerships are directed only to those in those of the opposite gender in the same sexual activity group as their own. The remaining fraction $1-\varepsilon$ are shared randomly with those in the opposite gender, according to the number of partnerships available. For men and women, $\rho_{k,l,k',l'}$ is the fraction of partnerships individuals in the kth gender and l^{th} activity-group form with individuals of the opposite gender of the k'^{th} gender and l'^{th} activity group:

Individuals with AIDS or failed ART are assumed to be not sexually active. $W_{k,l}$ is the total number of sexual partnership offered by individuals in that gender and sexual activity group:

$$W_{k,l} = c_{k,l} \sum_{s=1,2,3,4,6} X_{k,l}^{s}$$

For women (k=1, k'>1): $\rho_{1,l,k',l'} = \varepsilon \delta_{l,l'} \frac{W_{k',l'}}{\sum_{k'=2}^{4} W_{k',l'}} + (1-\varepsilon) \frac{W_{k',l'}}{\sum_{l'} \sum_{k'=2}^{4} W_{k',l'}}$

For men (k>1): $\rho_{k,l,k',l'} = \varepsilon \delta_{l,l'} + (1-\varepsilon) \frac{W_{1,l'}}{\sum_{l'} W_{1,l'}}$

Here, $\delta_{i,j}$ is the Kronecker delta: $\delta_{ij} = \begin{cases} 1, & \text{if } i = j \\ 0, & \text{if } i \neq j \end{cases}$

To ensure that the total number of partnerships formed by men (circumcision status *a*, risk-group *b*) and women (risk-group *d*) are consistent, the following correction is made:

$$D_{a,b,c} = \frac{W_{a,b} c_{a,b} \rho_{a,b,1,c}}{W_{1,c} c_{1,b} \rho_{1,c,a,b}}$$

$$\rho_{a,b,1,c} \rightarrow \rho_{a,b,1,c} \left(D_{a,b,c}\right)^{\theta-1}$$

$$\rho_{1,c,a,b} \rightarrow \rho_{1,c,a,b} \left(D_{c,a,b}\right)^{\theta}$$

In this way, θ determines the extent to which the pattern of partnership formation is determined by the parameters estimated from men's reported sexual behaviour.

In a partnership between individuals in the in risk groups *l* and *l'*, the number of sex acts and the level of condom use is determined by whether the partnership is classified as 'regular' or 'casual'. If the sum of risk groups of the partners is less than 4, the partnership is classified as 'casual', otherwise it classified as 'regular'. The scheme used to decide this is tabulated below:

		Partner's risk group (l')		
		**1 (highest risk)**	**2**	**3 (lowest risk)**
Own risk group (l)	**1 (highest risk)**	Casual (*l*+*l'*=2)	Casual (*l*+*l'*=3)	Regular (*l*+*l'*=4)
	**2**	Casual (*l*+*l'*=3)	Regular (*l*+*l'*=4)	Regular (*l*+*l'*=5)
	**3 (lowest risk)**	Regular (*l*+*l'*=3)	Regular(*l*+*l'*=5)	Regular (*l*+*l'*=6)

Table 1: Classification of casual and regular partnerships.

For convenience, we write $L = l + l'$. This classification determines the number of sex acts in each partnerships (n_L) and the fraction of partnerships in which condoms are used consistently and correctly throughout ($d_{L,q}$). For the latter, *q* indicates whether the male partner is circumcised (*q*=1) or not (*q*=0).

The force of infection is calculated as:

$$\lambda_{1,l} = c_{1,l} \sum_{k'=2}^{4} \sum_{l'} \left((1 - d_{L,q}) \rho_{1,l,k',l'} \left(1 - (1 - p_{k',l'})^{n_L}\right) \right) \quad (\mathrm{q} = 0 \text{ if } \mathrm{k'} = 2; \mathrm{q} = 1 \text{ otherwise})$$

$$\lambda_{k,l} = c_{k,l} \sum_{l'} \left((1 - d_{L,q}) \rho_{1,l,1',l'} \left(1 - (1 - p_{1,l'})^{n_L}\right) \right) \text{ for k} > 1 \quad (\mathrm{q} = 0 \text{ if } \mathrm{k} = 2; \mathrm{q} = 1 \text{ otherwise})$$

Here, $p_{k',l'}$ is the average chance of transmission per sex-act in a sexual partnerships with individuals of that gender and risk-group. It depends on the prevalence and the stage of infection of those in that group in the following way:

$$p_{k',l'} = \frac{\sum_{s=1,2,3,4,6} \beta_s X^{s}_{k',;'}}{W_{k',l'}}$$

(iv) Parameterisation

Default values for all parameters are given in Table 1. Sexual behaviour parameters are based on published observations in rural Eastern Zimbabwe [1], where possible. HIV transmission probabilities are informed from cohort studies of discordant couples in Uganda [2]. Figure 1 shows the simulated course of the HIV epidemic using the default parameters, which is good agreement with other models and measurements of prevalence in national surveillance systems [3]. Population growth rate pre-AIDS and in the AIDS-era are in good agreement with empirical observations in rural Zimbabwe [4].

<table>
<tr><th>Parameter Symbol</th><th>Meaning</th><th>Value</th></tr>
<tr><td>$\phi_{k,l}$</td><td>Fraction entering each sexual activity risk-group</td><td><table><tr><td></td><td>Women (k=1)</td><td>Men (k=2)</td></tr><tr><td>l=1</td><td>0.1</td><td>0.1</td></tr><tr><td>l=2</td><td>0.3</td><td>0.3</td></tr><tr><td>l=3</td><td>0.6</td><td>0.6</td></tr></table></td></tr>
<tr><td>$1/\mu$</td><td>Mean duration sexually active</td><td>40 years</td></tr>
<tr><td>α</td><td>Population growth rate in absence of AIDS</td><td>2.5% per year</td></tr>
<tr><td>$1/\sigma_s$</td><td>Mean duration with each stage of HIV infection</td><td><table><tr><td>s=1 (acute)</td><td>4 months</td></tr><tr><td>s=2 (latent)</td><td>8 years</td></tr><tr><td>s=3 (pre-AIDS)</td><td>12 months</td></tr><tr><td>s=4 (AIDS)</td><td>6 months</td></tr><tr><td>s=5 (Treatment)</td><td>8 years</td></tr><tr><td>s=6 (failed treatment)</td><td>3 months</td></tr></table></td></tr>
<tr><td>R</td><td>Replacement of high-risk groups (R=0 means no replacement; R=1 means replacement) – see text for details</td><td>1</td></tr>
<tr><td>$a(t)$</td><td>Fraction of individuals developing AIDS that start ART.</td><td><table><tr><td>$a_{\max}$</td><td>0.9</td></tr><tr><td>a_{rate}</td><td>0.215</td></tr><tr><td>a_{start}</td><td>Epidemic year 25</td></tr></table></td></tr>
<tr><td>$v(t)$ and $g(t)$</td><td>Rate of adults being circumcised and fraction of men starting sex that are already circumcised</td><td><table><tr><td>τ</td><td>1 year</td></tr><tr><td>$v_{\max}$</td><td>Varied to generate different coverage levels</td></tr><tr><td>$g_{\max}$</td><td>Varied to generate different coverage levels</td></tr></table></td></tr>
<tr><td>T_{HIV}</td><td>Relative rate of being</td><td>1</td></tr>
</table>

	circumcised if infected with HIV with reference to those not infected. (1 implies same rate, i.e. random)	
T_{RISK}	Relative rate of being circumcised if in top two risk-groups, with reference to those in lowest risk-group. (1 implies same rate, i.e. random).	1
$1/w$	Duration of wound healing following circumcision	2 months
c_k	Mean rate of partner change	Women (k=1): 3<br>Men (k=2): 3<br>Wound-healing men (k=3): bc_2<br>Circumcised men (k=4): bc_2
b	Proportional change in mean rate of partner change rate following circumcision (1 implies no change).	1
$\varpi_{k,l}$	Relative rate of partner change in the risk-groups, with respect to the lowest risk-group.	(columns: Women (k=1) / Men (k>1))<br>l=1: 100 / 100<br>l=2: 20 / 20<br>l=3: 1 (definition) / 1 (definition)
β_s	HIV transmission probability (per sex-act).	s=1: 0.01<br>s=2: 0.001<br>s=3: 0.005<br>s=4: n/a<br>s=5: 0.00001<br>s=6: n/a
n_L	Number of sex acts in partnerships. *L=l'+l* (the sum of the risk-group of both partners).	L(=l'+l)<4: 25<br>L(=l'+l)≥4: 100
$d_{L,q}$	Fraction of partnerships in which condoms are used consistently throughout.	(columns: Man not circumcised (*q*=0) $d_{L,0}v$ / Man circumcised (*q*=1) $d_{L,1}v$)<br>L(=l'+l)<4: 0.6 / 0.6*v*

		L(=l'+l)≥4	0.35	0.35
v	Change in condom use with casual partners following circumcision (1 implies no change).	1		

Table 1: Default parameter values (in the order they appear in the text).

(v) Model outputs

Overall incidence rates in the simulations is calculated as; $inc(t) = \sum_k \sum_l \lambda_{k,l} X^0_{k,l}$. (For ease of reading, we have not written that $\lambda_{k,l}$ and $X^s_{k,l}$ are both functions of time, *t*, as well).

The incidence rate ratio (IRR) is calculated by running the simulation with and without the intervention, and comparing the derived incidence time-series: $IRR(t) = \frac{[inc(t)]_{itnv}}{[inc(t)]_{baseline}}$.

The IRR disaggregated by gender and circumcision status is adjusted to prevent confounding through, for example, circumcised men being disproportionately high-risk or infected, or different patterns of infection leading to differential depletion of the risk-groups over in the baseline and intervention scenarios. The risk-distribution adjusted-IRR for the *k*th gender is denoted $aIRR_k(t)$:

$$aIRR_k(t) = \frac{\sum_l [\lambda_{k,l}]_{\text{int}\,v} [X_{k,l}]_{\text{int}\,v}}{\sum_l [\lambda_{k,l}]_{baseline} [X_{k,l}]_{\text{int}\,v}}$$

References

1. Gregson S, Nyamukapa CA, Garnett GP, Mason PR, Zhuwau T, et al. (2002) Sexual mixing patterns and sex-differentials in teenage exposure to HIV infection in rural Zimbabwe. Lancet 359: 1896-1903.
2. Wawer MJ, Gray RH, Sewankambo NK, Serwadda D, Li X, et al. (2005) Rates of HIV-1 transmission per coital act, by stage of HIV-1 infection, in Rakai, Uganda. J Infect Dis 191: 1403-1409.
3. UNAIDS, WHO (2007) AIDS Epidemic Update. (available from http://dataunaidsorg/pub/EPISlides/2007/2007_epiupdate_enpdf).
4. Gregson S, Nyamukapa C, Lopman B, Mushati P, Garnett GP, et al. (2007) Critique of early models of the demographic impact of HIV/AIDS in sub-Saharan Africa based on contemporary empirical data from Zimbabwe. Proc Natl Acad Sci U S A 104: 14586-14591.

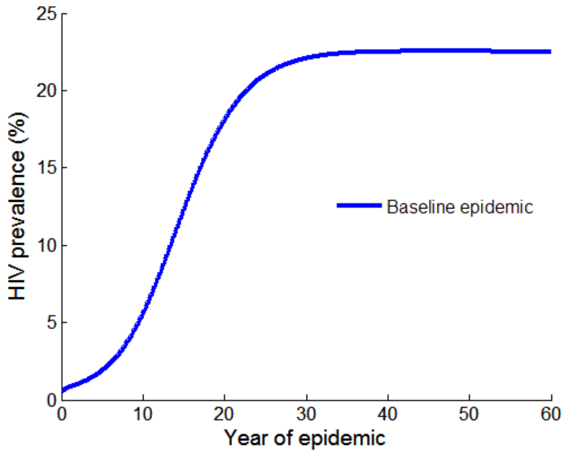

Figure 1: Simulated HIV prevalence over time when no intervention is simulated.
